# Development of an Automatic Identification System Autonomous Positioning System

**DOI:** 10.3390/s151128574

**Published:** 2015-11-11

**Authors:** Qing Hu, Yi Jiang, Jingbo Zhang, Xiaowen Sun, Shufang Zhang

**Affiliations:** 1 Information Science and Technology College, Dalian Maritime University, Dalian 116026, China; E-Mails: hq0518@dlmu.edu.cn (Q.H.); zhang_jingbo@dlmu.edu.cn (J.Z.); sunny@dlmu.edu.cn (X.S.); sfzhang@dlmu.edu.cn (S.Z.); 2 School of Electronic and Information Engineering, Beihang University, Beijing 100083, China

**Keywords:** positioning system, automatic identification system (AIS), ranging-mode, additional secondary factor (ASF)

## Abstract

In order to overcome the vulnerability of the global navigation satellite system (GNSS) and provide robust position, navigation and time (PNT) information in marine navigation, the autonomous positioning system based on ranging-mode Automatic Identification System (AIS) is presented in the paper. The principle of the AIS autonomous positioning system (AAPS) is investigated, including the position algorithm, the signal measurement technique, the geometric dilution of precision, the time synchronization technique and the additional secondary factor correction technique. In order to validate the proposed AAPS, a verification system has been established in the Xinghai sea region of Dalian (China). Static and dynamic positioning experiments are performed. The original function of the AIS in the AAPS is not influenced. The experimental results show that the positioning precision of the AAPS is better than 10 m in the area with good geometric dilution of precision (GDOP) by the additional secondary factor correction technology. This is the most economical solution for a land-based positioning system to complement the GNSS for the navigation safety of vessels sailing along coasts.

## 1. Introduction

Highly reliable and robust position, navigation and time (PNT) data is at the core of shipboard and shore-based electronic systems using the Worldwide Radio Navigation System (WWRNS) of the International Maritime Organization (IMO). The Global Navigation Satellite System (GNSS) is the primary PNT information provider nowadays. IMO has recently become aware of the vulnerability of GNSS to both naturally occurring and man-made interferences [1,2]. Thus, in order to guarantee coastal navigation safety, an alternative backup position system to complement the existing GNSS is recommended.

The various position systems used in marine navigation have been widely investigated in the literature, such as e-Loran systems [3,4,5,6], inertial navigation systems [7,8], terrain referenced navigation systems [9], *etc.* However, each of all these position systems is an independent navigation system. The existing Automatic Identification System (AIS) is a communication system for maritime information transition, such as Maritime Mobile Service Identity (MMSI), position, course, and speed. AIS base stations have been already located along coastlines of IMO’s member countries, and shipborne AIS equipment is mandatory on most vessels according to the IMO requirements [10]. However, the position information in the AIS equipment typically originates from a GNSS receiver. If the AIS could provide PNT information by itself, not depending on the other navigational sensors, it could significantly promote the robustness of the PNT. Furthermore, there would be no need to establish new reference stations for positioning and load new positioning equipment on vessels. Thus, the costs of the construction and maintenance of the reference stations could be saved, and the vessels would not need to be equipped with the other land-based navigation aids. It is an economical solution for the land-based positioning system to complement the GNSS for the navigation safety of vessels sailing along the coast. Therefore the development and trials of ranging-mode using MF and AIS signals to enhance marine navigation into the WWRNS is encouraged [11].

In this paper, the use for coastal navigation and maritime safety of a position system based on ranging-mode AIS, called the AIS Autonomous Position System (AAPS), is investigated. Based on the coastal AIS base stations established all over the world, autonomous positioning is achieved by measuring very high frequency (VHF) radio signals from AIS base stations to the shipborne AIS equipment, which is mandatorily installed according to the IMO norms. The principle of the AAPS is investigated, including the position algorithm, the signal measurement technique, the geometric dilution of precision (GDOP), the time synchronization technique and the additional secondary factor (ASF) correction technique. In the AAPS, the AIS base stations are time synchronized. The shipborne AIS can measure the transmission delay of the VHF AIS signal from the AIS base stations. Then the ASF correction is used to reduce any signal propagation errors. Finally, the vessel location can be estimated according to the position algorithm based on the signal propagation time. Based on the above research, a verification system for the AAPS has been established. The original function of AIS base stations has not been affected. Static and dynamic experiments have been carried out to verify the positioning function. The experimental results show the static positioning accuracy is 9.834 m (2*σ*) and the dynamic positioning accuracy is 9.996 m (2*σ*) with GDOP of less than 1.5.

The rest of the paper is organized as follows: Section 2 discusses the principle of the AAPS, including the position algorithm, the signal measurement technique, the GDOP, the time synchronization technique and the ASF correction technique. The proposed AAPS verification system in the Xinghai sea region of Dalian (China) is established and the system’s experimental scheme is given in Section 3. Static and dynamic experiments performed to verify the position function of AAPS and the experimental results are discussed in Section 4. Finally, some conclusions are put forth in Section 5.

## 2. Principle of the AIS Autonomous Positioning System

**Figure 1:**
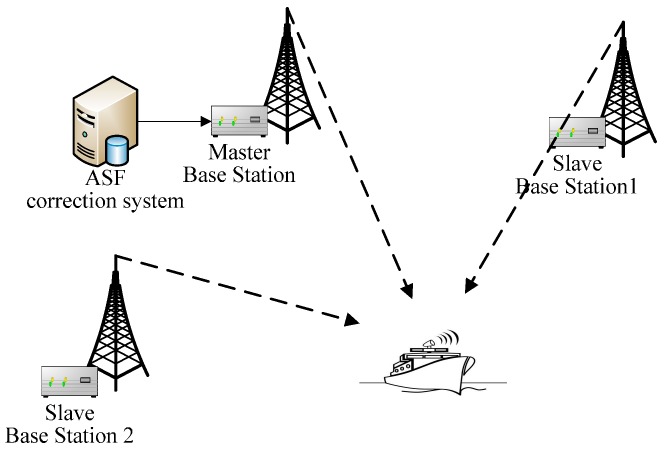
AIS autonomous positioning system configuration.

The AAPS is comprised of a master AIS base station (BS), some slave base stations, a shipborn AIS equipment and an ASF correction system. The system configuration is shown in Figure 1. The AIS BSs require time synchronization. The master AIS BS and other slave BSs takes turns sending signals. The shipborn AIS equipment receives the VHF AIS signal from the BSs. Furthermore, an ASF correction system is set up in order to improve the positioning accuracy. In this section, the principle of AAPS is investigated in detail.

### 2.1. Position Algorithm

The ranging—mode position technique in AIS is based on a signal propagation delay technique. Signal propagation time position techniques can be categorized as time of arrival (TOA) [12] and time difference of arrival (TDOA) [13].

In the TOA technique, the fundamental objective is to estimate the vessel position by measuring the signal transmission delay from BSs to the vessel. Then the distance can be obtained by multiplying by the speed of light in the free space *c*. However, in practice deviations exist in the calculated distance values due to the clock bias between the vessel and the AIS BSs. Thus the positioning equation used for the TOA technique should be written as:

(1) $$\bar{R_{i}}=\sqrt{{\left(x_{i}-x\right)}^{2}+{\left(y_{i}-y\right)}^{2}}+c\cdot \Delta t$$

where $\bar{R_{i}}$ is the measured distance from the *i*th BS to the vessel; (*x*, *y*) is the unknown true position of the vessel; (*x_i_*, *y_i_*) is the known precise position of the *i*th BS; Δ*t* is the clock bias between the vessel and the BSs. The clock bias augments the two-dimensional location vector forming a three dimensional state vector, so there are three unknowns (*x*, *y* and Δ*t*) in the above equation. Thus, to estimate the vessel position, more than three independent TOA measurements are required at the same time from the different BSs. Otherwise, there are only two channels in the shipborn AIS equipment, so it is very difficult to get more than three measurements at the same time.

In the TDOA technique, the vessel position is estimated by measuring the relative time delay between the master and the slave BSs. This does not require knowledge of the precise absolute time that the signal left the BS. Thus, the vessel doesn’t need to synchronize with the BSs, so it is more feasible for users in practice. The positioning equation used for the TDOA technique is given by:

(2) $$\Delta R_{i}=\sqrt{{\left(x_{i}-x\right)}^{2}+{\left(y_{i}-y\right)}^{2}}-\sqrt{{\left(x_{m}-x\right)}^{2}+{\left(y_{m}-y\right)}^{2}}$$

where (*x_i_*, *y_i_*) and (*x_m_*, *y_m_*) are the known precise coordinates of the *i*th slave BS and the master BS, respectively. As there are two unknowns in the position equation, at least two BS couples are required for the distance difference measurements at the same time.

Yi [14,15] analysed the mean square error for potential position algorithms based on the ranging-mode AIS used in AAPS. The performance of the TDOA technique is always superior to the TOA technique without time synchronization. Thus, the positioning algorithm for AAPS uses the TDOA technique. The details of the position estimation algorithm based TDOA technique in AAPS is presented in [16].

### 2.2. Signal Measurement Technique

**Figure 2:**
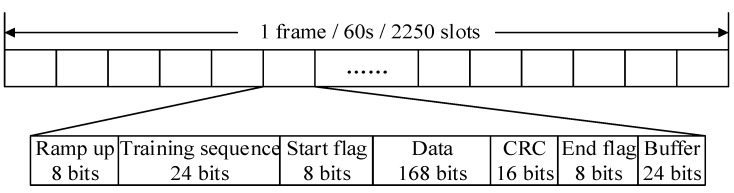
Structure of AIS signals.

In the existing AIS, the maritime information is transmitted in the VHF mobile band. Specifically, two channels located at 161.975 and 162.025 MHz have been designated for this purpose [17]. The two channels organized into time slots are shared by all BSs using time division multiple access (TDMA). Each channel has 2250 slots per minute. The data signaling rate is 9600 bits per second. The structure of the AIS signal is illustrated in Figure 2.

**Figure 3:**
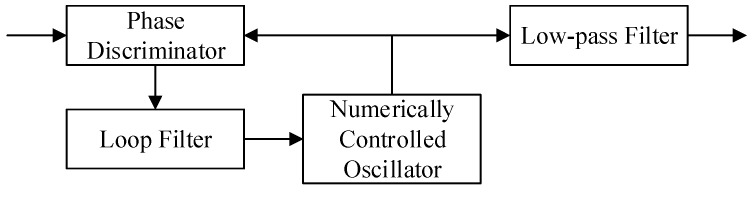
Block diagram of GMSK demodulation in an AIS receiver.

The non-coherent demodulation technique is widely used in traditional shipborne AIS receivers for communication. There is no strict requirement for the decision time of each bit, thus the measurement accuracy of the bit transition is theoretically one bit period. This cannot meet the accuracy requirements of the positioning system, so the carrier frequency and the phase of the AIS signals from BSs should also be tracked in the receiver for AAPS. However, the problem of the cycle ambiguity is difficult to resolve, as the carriers of the AIS signals are not the continuous wave signals. The carrier-phase measurement alone is not feasible. The receivers used in AAPS estimate the carrier frequency, the carrier phase and the time of bit transition. Then they can provide precise times for the bit transitions with assistance of carrier tracking. The corresponding block diagram is shown in Figure 3. The method is called GMSK demodulation based on carrier phase tracking. The key technology used in this demodulation method is the carrier extraction and tracking, which has been investigated in [18].

**Figure 4:**
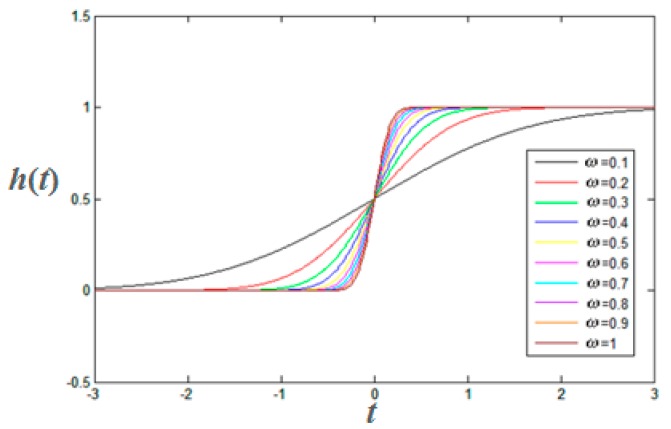
Step response of a Gauss filter.

GMSK is a noncausal system [19]. The step response function of Gauss filter is given as:

(3) $$h(t)=\frac{1}{2}\times \left[1+erf(\frac{\omega }{\sqrt{2\text{ln}2}}\times t)\right]$$

where *ω* denotes the bandwidth. The simulation curves with different *ω* are shown in Figure 4.

It can be seen from Figure 4 that the location of each zero crossing of the baseband signal corresponds to the location of the bit transition of serial data signals. Each zero crossing of the baseband signal can be detected in GMSK demodulation based on carrier phase tracking. Therefore, the precise transition time from one bit to the next bit in the serial data signals can be measured in GMSK demodulation based on a FPGA circuit.

In the AIS receiver based on FPGA, a special counter is used to record the bit transition time of serial data signals. It starts from zero at the beginning of a slot. However, the measurement error of the single measurement according to the counter cannot be ignored due to the existence of random errors and quantization errors. In order to reduce the measurement error of the single sampled value, a number of sampled values in one slot are measured. The first sampled value of time delay measurement corresponds to the last bit of the start flag (7E). After that, each bit is sampled in one time slot. There are 193 sample datapoints from the last bit of the start flag to the end flag in one time slot. The measurement of time delay *T_i_* corresponding to the *i*th bit is given by:

(4) $$T_{i}=(8+24+7+i)\cdot B+\Delta T=(39+i)\cdot B+\Delta T$$

where *B* is the time of one bit, which is calculated according to the bit synchronization signal of the AIS receiver; Δ*T* is the time delay from the BS to the vessel. 8, 24 and 7 are the lengths of the ramp up, the training sequence and the first seven bits of the start flag shown in Figure 2. The unit of these parameters in Equation (3) is the clock cycle count with a frequency of 174.72 MHz. The use of a clock frequency of 174.72 MHz is a compromise. The maximum frequency of the designed AIS receiver based on FPGA is less than 200 MHz. As the intermediate frequency of AIS signals is 455 KHz, the clock frequency should be the integer times of the intermediate frequency of AIS signal, so to obtain higher time resolution, 174.72 MHz is chosen as the clock frequency.

**Figure 5:**
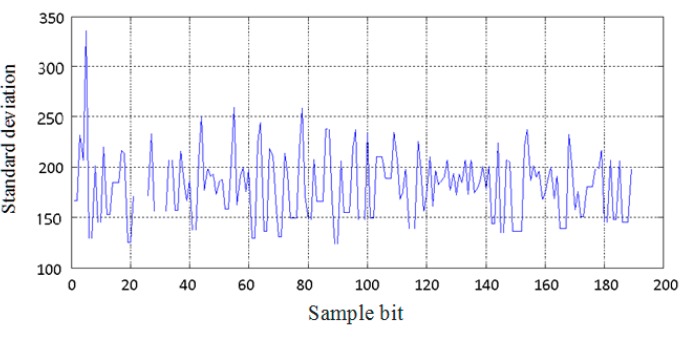
Standard deviation of measurement for sample data.

To evaluate the accuracy of the measurement, a total of 193 sample data in one slot is analyzed during 700 time slots. The curve of the standard deviation of time delay measurement according to every sample data is drawn in Figure 5. It can be seen that the standard deviation of the measurement corresponding to every sample bit is very large. The average reaches 179.9156 clock cycles count, corresponding to 1.04 µs, so the accuracy of the distance measurement is 312 m. It cannot meet the requirements of positioning accuracy.

**Figure 6:**
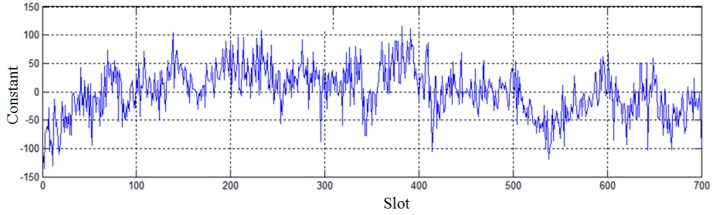
Experimental results after the linear fitting based on the least squares method.

Under the ideal condition, all of the time data on the transition from one bit to the next bit in a slot should be on a straight line, with a slope of the baud rate of replication synchronization bit. Thus, the least squares method is used for the linear fit of all of the time data on the transition from one bit to the next bit in a slot. The time of the bit transitions can be measured more accurately by reducing the random turbulence. According to the Equation (3), the first order coefficient after the linear fitting is the corrected value of the bit synchronization signal, and the constant is the estimation of the time delay. Experimental results after the linear fitting based on the least square method are shown in Figure 6. In Figure 6, the horizontal axis represents the number of the slot and the vertical axis represents the constant calculated by the linear fitting corresponding to each slot, which indicates the estimation of the time delay. The standard deviation of measurement corresponding to the time delay after the linear fitting is 42.70 clock cycle counts, corresponding to 0.24 µs, so the accuracy of the distance measurement is reduced to 73 m by the linear fitting based on the least squares method.

### 2.3. Geometric Dilution of Precision

Accuracy is a crucial parameter for evaluating a positioning system. The accuracy depends on the measurement accuracy and the geometry of the BSs, which is described by the GDOP [20]. In general, positioning errors can be expressed as the product of the GDOP at the vessel location and the root mean square deviation of the measurement errors [21]. The measurement error has been discussed above in Section 2.2. The GDOP of AAPS will be investigated below. The existing AIS BSs have already been established for a communication system, but the layout of the location of AIS BSs is not suitable for positioning, without considering the effects of the geometry, so we need to evaluate the GDOP of AIS BSs in AAPS.

The location equation (Equation (2)) is nonlinear. We obtain the linear location equation using a Taylor-series, keeping only terms below second order:

(5) $$\Delta {\bar{R}}_{i}=\Delta {\hat{R}}_{i}+\frac{\partial \Delta {\hat{R}}_{i}}{\partial x}dx+\frac{\partial \Delta {\hat{R}}_{i}}{\partial y}dy$$

where $\Delta {\bar{R}}_{i}$ and $\Delta {\hat{R}}_{i}$ denote the measurement value and its estimation for the *i*th pair of BSs, respectively. Stacking all the measurements from different BSs, the above Equation (4) can be written in matrix form as follows:

(6) $$\left[\begin{array}{c} \delta R_{1}\\ \vdots \\ \delta R_{n} \end{array}\right]=\left[\begin{array}{cc} \frac{\partial \Delta {\hat{R}}_{1}}{\partial x} & \frac{\partial \Delta {\hat{R}}_{1}}{\partial y}\\ \vdots & \vdots \\ \frac{\partial \Delta {\hat{R}}_{n}}{\partial x} & \frac{\partial \Delta {\hat{R}}_{n}}{\partial y} \end{array}\right]\left[\begin{array}{c} dx\\ dy \end{array}\right]$$

where:

(7) $$\begin{array}{l} \begin{array}{c} \alpha_{i}=\frac{\partial \Delta {\hat{R}}_{i}}{\partial x}=\frac{x_{m}-x}{{\hat{R}}_{m}}-\frac{x_{i}-x}{{\hat{R}}_{i}}=-\text{cos}\gamma_{m}+\text{cos}\gamma_{i}\\ \beta_{i}=\frac{\partial \Delta {\hat{R}}_{i}}{\partial y}=\frac{y_{m}-y}{{\hat{R}}_{m}}-\frac{y_{i}-y}{{\hat{R}}_{i}}=-\text{sin}\gamma_{m}+\text{sin}\gamma_{i} \end{array}\\ \delta R_{i}=\Delta {\bar{R}}_{mi}-\Delta {\hat{R}}_{mi} \end{array}$$

where *γ*_i_ is the estimated azimuth angle directed from the estimated position of the vessel to the *i*th BS. As:

(8) $$G=\left[\alpha \beta \right]=\left[\begin{array}{ccc} -\text{cos}\gamma_{m}+\text{cos}\gamma_{1} & & -\text{sin}\gamma_{m}+\text{sin}\gamma_{1}\\ \vdots & & \vdots \\ -\text{cos}\gamma_{m}+\text{cos}\gamma_{n} & & -\text{sin}\gamma_{m}+\text{sin}\gamma_{n} \end{array}\right]$$

According to Equation (8), then the GDOP for AAPS can be calculated by:

(9) $$GDOP={\left(G^{T}G\right)}^{-1}$$

We take the established verification system of AAPS as an example in this paper. The location information of BSs in the verification system is shown in Table 1.

**Table 1:** Location information of base stations.

BS Name	Latitude	Longitude
Lingjing	38°50'21.309"N	121°30'46.005"E
Heishijiao	38°51'58.689"N	121°33'05.779"E
Fujiazhuang	38°51'52.660"N	121°36'49.543"E

**Figure 7:**
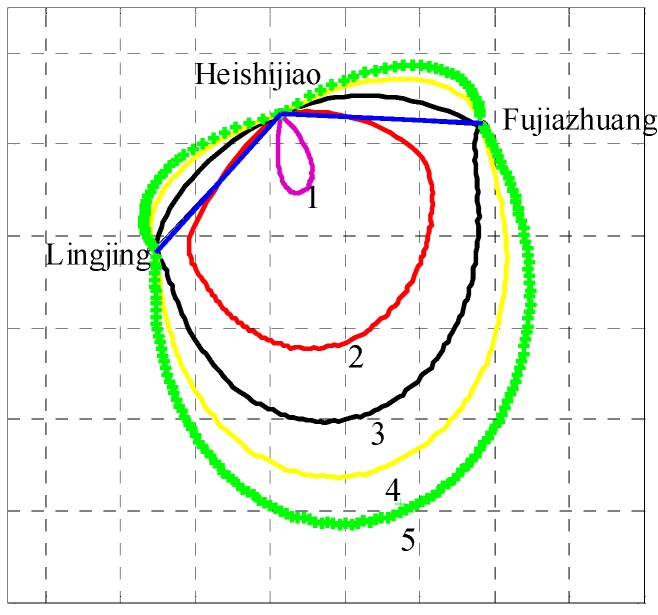
GDOP for the verification system of AAPS.

The GDOP of the established verification system of AAPS is evaluated as shown in Figure 7. GDOP is calculated based on the vessel’s bearings to each of the BSs. Since the GDOP can be interpreted as a multiplier on the measurement error, lower numbers are better. According to the conclusions of Section 2.2, the accuracy of the distance measurement is 73 m by the linear fitting based on the least squares method, therefore, the positioning precision for the verification system of AAPS is about 100 m (2*σ*) in the area where GDOP is less than 1.5.

### 2.4. Time Synchronization Technique

The most prominent advantage of the TDOA technique is that the vessel doesn’t need to synchronize with the BSs. This is more feasible for users in reality. Even so, it still requires synchronization between all the BSs. As part of their definition as a TDMA communications link, AIS transmissions are synchronized. The BSs are synchronized with the coordinated universal time (UTC) given by the GNSS receivers. According to the time synchronization specification in IEC 62320-1 [22], the AIS BS has a time source synchronization to UTC better than 50 µs. This obviously cannot meet the accuracy requirement for the position system.

Time synchronization in AAPS could be achieved using the GNSS disciplined rubidium clock technology [23]. The principle of the GNSS disciplined rubidium clock technology is described briefly below. The phase difference between pulse per seconds (PPS) given by the rubidium clock and the GNSS time receiver is compared in real-time. According to the phase difference, the phase locked loop adjusts the rubidium clock frequency, which is used for time synchronization of the BSs. The technology of GNSS disciplined rubidium clock accomplishes a couple of things: One is making the rubidium clock output frequency of 10 MHz more accurate. The other is making the PPS output by the rubidium clock more accurate and stable, which is aligned with the PPS output from the GNSS time receiver.

When the phase of the PPS output by the rubidium clock is aligned to the PPS output from the GNSS receiver, it’s called synchronization mode. It could be used to provide the time synchronization up until the point in time that the GNSS signal is lost. When the GNSS signal is not present, it is called holdover mode. After this point, the signal would remain in tolerance for 24 h for a rubidium clock in the holdover mode. When the GNSS signal is received again, it can quickly resume the high precision synchronization.

**Figure 8:**
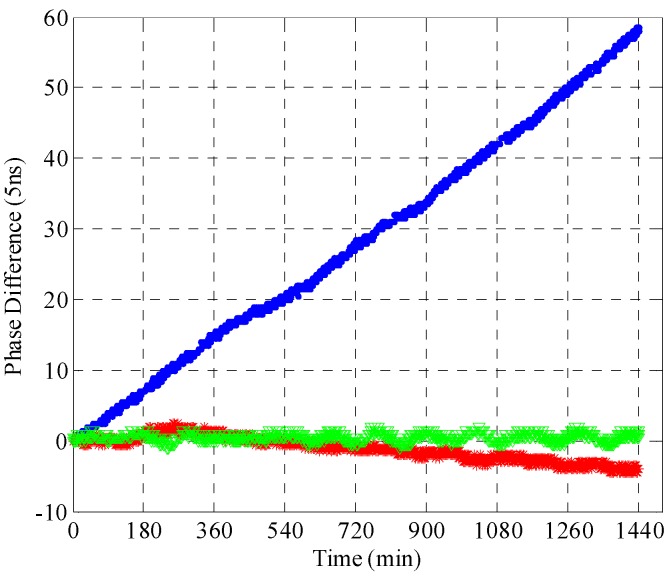
Experimental results of GNSS disciplined rubidium clock technology.

In the experiment, the rubidium clock had been locked to the GNSS time receiver for the first 2 h, but lost it later. Figure 8 illustrates the experimental results in the holdover mode for 24 h, in which each division along the horizontal axis corresponds to one minute, and each division along the vertical axis corresponds to 5 ns.

When the rubidium clock is not locked to any reference, such as the GNSS time receiver, the phase difference between the PPS outputted by the rubidium clock and the GNSS receiver is changing with time as denoted by the blue dots in Figure 8. The frequency stability of the rubidium clock is influenced by environmental factors, especially the temperature. Thus, the laboratory must meet strict work environment requirements. The phase deviation is less than 300 ns within 24 h when the rubidium clock is running individually. This is consistent with performance levels given by [24]. The phase differences are represented by the green and the red dots in the synchronization mode and the holdover mode of GNSS disciplined rubidium clock technology, respectively. In the synchronization mode, the PPS given by the rubidium clock is always aligned with the PPS output from the GNSS time receiver. Therefore, the phase difference of a GNSS disciplined rubidium clock is very small. In the holdover mode, the rubidium clock has already been disciplined by GNSS. Although without the PPS output from the GNSS time receiver, the accurate frequency drift correction model can be used, whose parameters are obtained according to long-term statistics of the character of the rubidium clock and short-term statistics derived from synchronization mode data. The experimental results in Figure 8 show that the phase difference is less than 30 ns within 24 h in the holdover mode using the GNSS disciplined rubidium clock technology. However, the period of validity of short-term statistical data in the correction model is 24 h. The phase error will increase quickly after 24 h.

### 2.5. Additional Secondary Factor Correction

**Figure 9:**
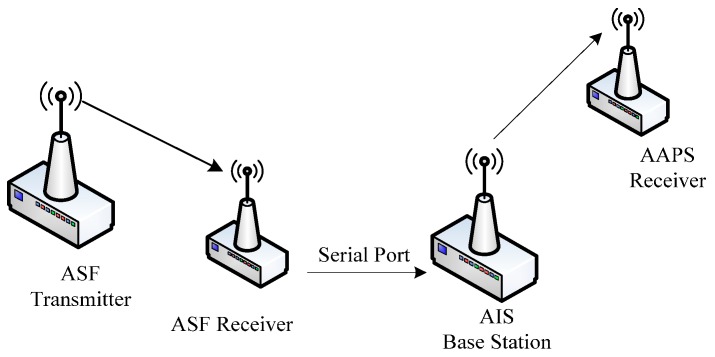
Connection diagram between the ASF correction system and AAPS.

Due to the signals propagation over paths of varying conductivity, topography and weather, there is a significant factor limiting the accuracy of the positioning system. Thus, in the ground-based positioning system, ASF correction is generally employed. In the investigation of ASF corrections, the simplest approach is a theoretical model construction, but in that case the calculation error is large and it cannot meet the accuracy requirements of AAPS. Thus, the ASF correction system has been set up [25]. In the ASF correction system, the ASF transmitter is time synchronized with the ASF receiver. The ASF transmitter sends its precise position and ranging code to the ASF receiver. Therefore, the theoretical distance *R*_ASF0_ from the ASF transmitter to the receiver can be calculated according to the precise position information of the ASF transmitter and the ASF receiver. The actual measurement distance *R*_ASF_ can be obtained by measuring the propagation time of signals from the ASF transmitter to the ASF receiver. The ASF correction value is calculated by comparing the distance error between the theoretical and actual measurement distance from the ASF transmitter to the receiver. Then the real-time correction value is broadcasted to the vessels in the area by the AIS base station to improve the positioning accuracy. The ASF correction system is connected with AAPS as shown in Figure 9.

The measurement variances of ASF and AIS receiver are denoted by $\sigma_{\text{ASF}}^{2}$ and $\sigma_{\text{AIS}}^{2}$, respectively. The theoretical and actual measurement distances from the AIS BS to the vessel are denoted by *R*_AIS0_ and *R*_AIS_, respectively. The relationship between them is given by:

(10) $$\frac{R_{ASF}-R_{ASF0}}{R_{AIS}-R_{AIS0}}=\frac{\sigma_{ASF}}{\sigma_{AIS}}\cdot \frac{R_{ASF0}}{R_{AIS0}}$$

where *R*_ASF_, *R*_ASF0_ and *σ*_ASF_ obtained by the ASF correction system are all sent to the AIS master BS through a serial port according to the interface protocol. The AIS BS sends these parameters to the vessels in the same area. The AAPS receiver corrects the measured distance *R*_AIS_ according to Equation (11) using these ASF parameters and its own *σ*_AIS_:

(11) $$R_{AIS0}=R_{AIS}-[(R_{ASF}-R_{ASF0})\frac{\sigma_{AIS}}{\sigma_{ASF}}\cdot \frac{R_{AIS}}{R_{ASF}}]$$

## 3. Establishment of an AIS Autonomous Positioning System

### 3.1. Layout of the AAPS Base Station

**Figure 10:**
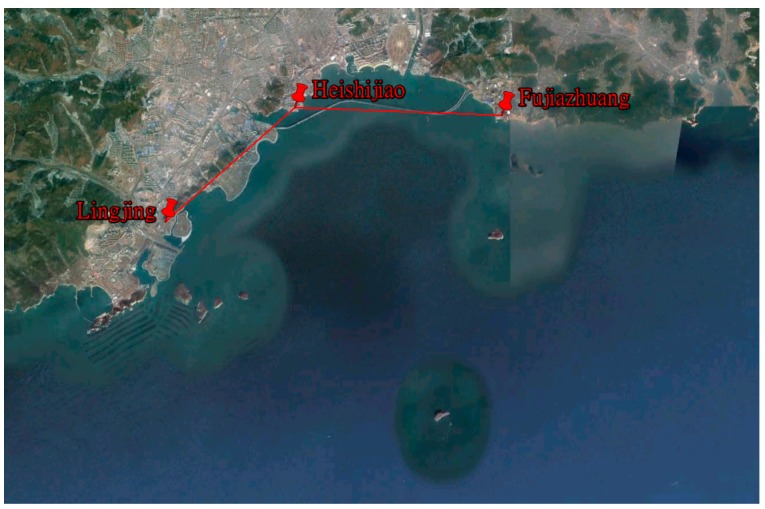
Location distribution of base stations in the verification system.

**Figure 11:**
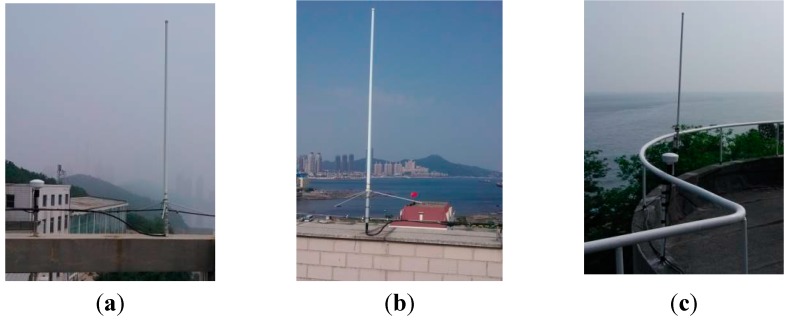
Working environment of the base stations: (**a**) Lingjing; (**b**) Heishijiao; (**c**) Fujiazhuang.

In order to verify the real-time positioning function of AAPS in a real sea area, an AAPS verification system was established, including a master BS and two slave BSs. If the layout of AAPS BSs is considered, the following factors should be taken into account: firstly, in order to ensure stable signal propagation, the BSs should be set up at the location with a view of the open sea. Secondly, at least three BSs are needed to meet the requirements for positioning. Thirdly, the GDOP of three BSs should be considered for improving the positioning accuracy. Considering the above factors, the AAPS verification system has been established in the Xinghai sea region of Dalian, with the BSs located in Lingjing, Heishijiao and Fujiazhuang, respectively. The location distribution of BSs is shown in Figure 10. The working environment of each BS is shown in Figure 11.

### 3.2. Experimental Scheme

**Figure 12:**
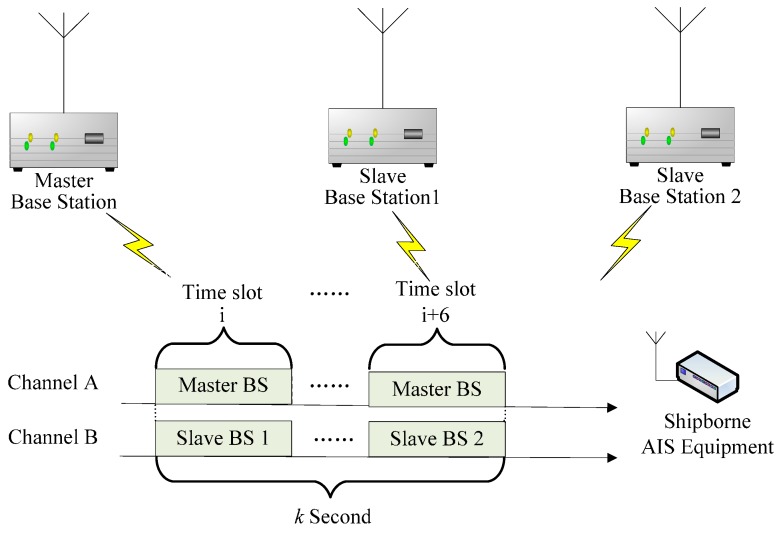
Experimental scheme of the verification system of AAPS.

The experimental scheme of the verification system of AAPS is shown in Figure 12. The BSs send signals according to the AIS slot regulations by time-sharing. Each AIS BS takes turns sending signals in a group according to its own MMSI. A master BS is included in every group. The master BS and the slave BS in the same group take turns sending signals in two channels (A and B) at the same time respectively. They start at zero seconds. The minimum transmission interval is one time slot, according to the protocol requirement of the radio communication sector of the ITU-R M.1371 [26]. Signals are sending repeatedly at every integral multiple of the positioning interval. The shipborne AIS equipment receives the VHF signals from the BSs, then measures the distance difference and estimates its position.

### 3.3. High Accuracy Reference System

In order to obtain more accurate position data as reference data to verify the performance of AAPS, the continuously operating reference station (CORS) system is employed. The DLMU-CORS system is designed and established in the Dalian coastal area. It can be used in the static and dynamic positioning accuracy tests of the AAPS.

**Figure 13:**
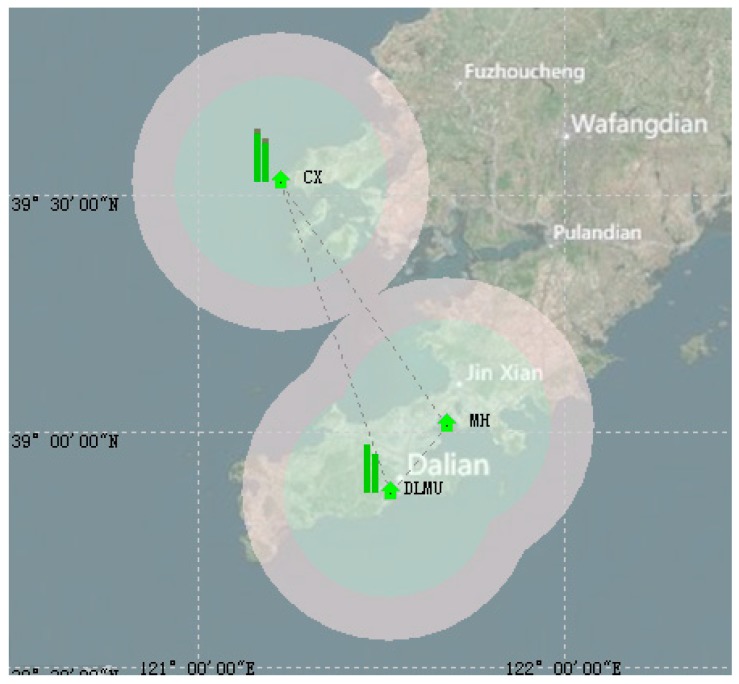
Signal coverage of DLMU-CORS.

The CORS system is a network of real time kinematic (RTK) base stations that broadcast corrections, usually over an Internet connection [27,28]. There are three base stations in the DLMU-CORS system, located at Dalian Maritime University, Mianhuang Island and Changxing Island. The coverage of signals is shown in Figure 13. It can be seen that the DLMU-CORS system can provide positioning reference data in the Dalian coastal area. The performance of the DLMU-CORS system can be evaluated the professional software—TopSURV. The dynamic positioning accuracy is 20 ms and the static positioning accuracy is 10 ms. Therefore, the DLMU-CORS system can satisfy the the signal coverage and the position accuracy requirements of AAPS performance evaluation.

## 4. Results and Discussion

**Figure 14:**
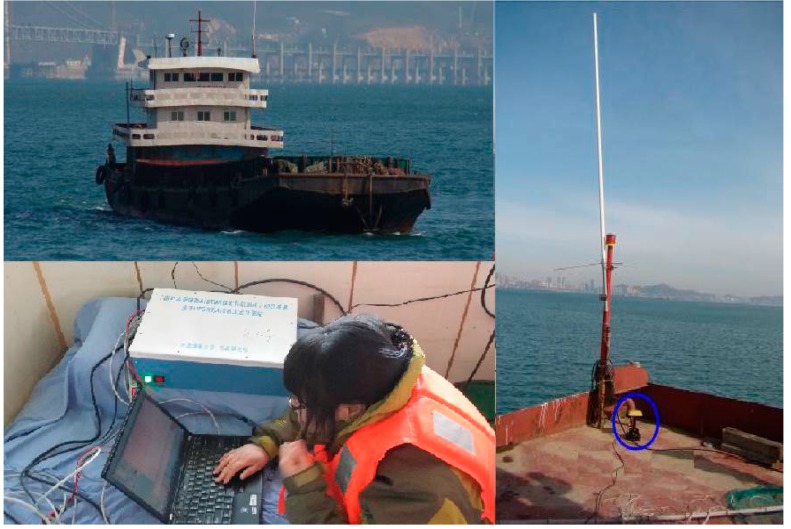
Positioning experiment scenario.

According to the experimental scheme, the static and dynamic positioning experiments of the verification system of AAPS are performed in an area where GDOP is less than 1.5. The estimated position of the vessel is compared with the high precision position provided by the DLMU-CORS system. The positioning accuracy of the verification system of AAPS is evaluated. The scenario of the position experiments is shown in Figure 14.

### 4.1. Static Positioning Experiment

**Figure 15:**
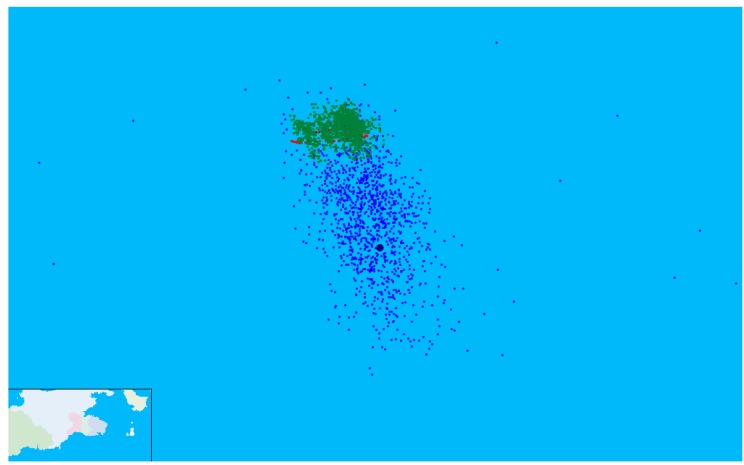
Real-time experimental results for the static position.

Positioning results of the shipborne AIS receiver for AAPS are collected for 86 min, while the vessel anchored in the area where GDOP is less than 1.5. Figure 15 shows the real-time positioning results displayed in the map. The precision position of the vessel given by the DLMU-CORS system was denoted by the red dots. We can see that the vessel was not absolutely static. There was a transverse swing when it was at anchor due to the influence of the wind and current. The positon results before using the ASF correction technology in the verification system of AAPS are indicated by the blue dots. It is obviously shown in Figure 15 that the latitude error is larger than the longitude error. The positon results after ASF correction are represented by the green dots. The position error has been decreased significantly using the ASF correction technology.

**Figure 16:**
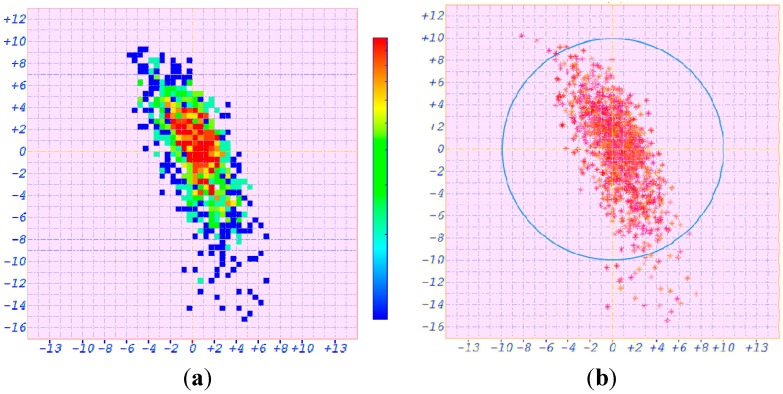
Positioning errors for the static position (**a**) Thermography diagram; (**b**) Scatter diagram.

The positioning error after ASF correction of the static positioning experiment is given in Figure 16. Figure 16a is the thermography diagram. The vertical axis denotes the latitude error. The mean and the root mean square (RMS) of the latitude errors are −0.667 and 4.039 m, respectively.

The longitude error is represented by the horizontal axis in Figure 16a. The mean and the RMS of the longitude error are +0.424 and 2.084 m, respectively. The position error scatter diagram is shown in Figure 16b. The radius of the blue circle is 10 m. Only 2.7% of all the position results are not in this blue positioning error circle. Thus the position error after ASF correction for the static position is 9.090 m (2*σ*).

### 4.2. Dynamic Positioning Experiment

**Figure 17:**
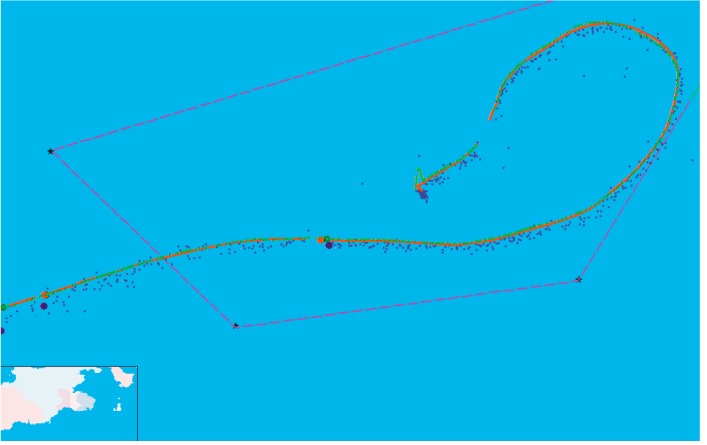
Vessel trajectory for the dynamic positioning experiment.

The dynamic position experiment for the verification system of AAPS was performed in the area where GDOP is less than 1.5. The trajectory of the vessel was drawn in Figure 17. The precision position originated from DLMU-CORS system was indicated by the red dots. The estimated positon of the vessel without using the ASF correction was denoted by the blue dots. The improvement of the positioning accuracy can be achieved by the ASF correction technology. Position results using the ASF correction were given by the green dots. The position error has been decreased significantly. The red dashed line represents the experimental range where GDOP is less than 1.5. The interruption of the vessel trajectory in Figure 17 was due to the temporary interruption of the power supply system caused by the switch between the vessel’s main and auxiliary generator. It can be seen that the positioning results without using the ASF correction technology are already able to characterize the trajectory of the vessel, but the position error is relatively large. The output trajectory for the verification system of AAPS using the ASF correction technology very nearly coincides with the vessel trajectory of the DLMU-CORS system.

**Figure 18:**
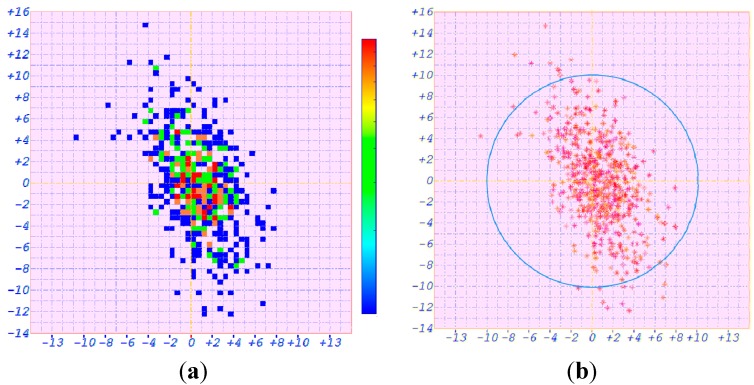
Positioning errors for the dynamic position (**a**) Thermography diagram; (**b**) Scatter diagram.

The positioning error of the dynamic positioning experiment in the verification system of AAPS is shown in Figure 18. The vertical axis denotes the latitude error and the horizontal axis represents the longitude error.

Figure 18a is the thermography diagram. The mean of the latitude error is −1.189 m and the RMS is 4.224 m. The mean and the RMS of the longitude error are +0.682 and 2.534 m, respectively. The position error scatter diagram is shown in Figure 18b. Only 3.5% of all the estimated position results are not in the blue positioning error circle, whose radius is 10 m. Thus the position error after ASF correction for the dynamic position is 9.851 m (2*σ*).

### 4.3. Discussion

The static and dynamic positioning experiments of the verification system of AAPS are performed in the area where GDOP is less than 1.5. The DLMU-CORS system is employed to obtain high accuracy reference data. The positioning accuracy of the verification system of AAPS is evaluated. The position errors after ASF correction for the static position and the dynamic position are 9.090 m (2*σ*) and 9.851 m (2*σ*), respectively. In conclusion, the positioning precision for the verification system of AAPS is about 100 m (2*σ*) in an area where GDOP is less than 1.5, and it can decrease to 10 m (2*σ*) using the ASF correction technology.

## 5. Conclusions

In order to overcome the vulnerability of the GNSS and provide robust PNT information in marine navigation, an autonomous positioning system based on ranging-mode AIS is presented in this paper. In the AAPS, the AIS base stations are all time synchronized using the GNSS disciplined rubidium clock technology. Then the shipborne AIS can get the transmission delay by measuring the precision time of the bit transitions with the assistance of carrier tracking. In order to reduce signal propagation errors, ASF correction is employed. Finally, the vessel location can be estimated according to the position algorithm based on the signal propagation time. In order to validate the proposed AAPS, a verification system has been established in the Xinghai sea region of Dalian. Static and dynamic positioning experiments were performed. The positioning precision of AAPS using the ASF correction technology is better than 10 m (2σ) in the area with good GDOP (less than 1.5). Based on the existing AIS, the position of the vessels can be estimated without establishing a new system. This is an economical solution for a land-based positioning system to complement the GNSS for the navigation safety of coastal vessels. In order to promote and popularize the AAPS, our future work will focus on the preformation improvement of the AAPS.
